# Association between an Anti-Inflammatory Dietary Score and Periodontitis—Evidence from the Population-Based Hamburg City Health Study

**DOI:** 10.3390/nu15143235

**Published:** 2023-07-21

**Authors:** Berit Lieske, Nina Moszka, Katrin Borof, Elina Larissa Petersen, Bettina Jagemann, Merle Ebinghaus, Thomas Beikler, Guido Heydecke, Ghazal Aarabi, Birgit-Christiane Zyriax

**Affiliations:** 1 Department of Periodontics, Preventive and Restorative Dentistry, Center for Dental and Oral Medicine, University Medical Center Hamburg Eppendorf, 20246 Hamburg, Germany; n.moszka@uke.de (N.M.); k.borof@uke.de (K.B.); t.beikler@uke.de (T.B.); g.aarabi@uke.de (G.A.); 2 Midwifery Science—Health Care Research and Prevention, Research Group Preventive Medicine and Nutrition, Institute for Health Service Research in Dermatology and Nursing (IVDP), University Medical Center Hamburg-Eppendorf, 20246 Hamburg, Germany; b.jagemann@uke.de (B.J.); m.ebinghaus@uke.de (M.E.); b.zyriax@uke.de (B.-C.Z.); 3 Population Health Research Department, University Heart and Vascular Center, University Medical Center Hamburg-Eppendorf, 20246 Hamburg, Germany; e.petersen@uke.de; 4 Department of Cardiology, University Heart and Vascular Center, University Medical Center Hamburg-Eppendorf, 20246 Hamburg, Germany; 5 Department of Prosthetic Dentistry, Center for Dental and Oral Medicine, University Medical Center Hamburg-Eppendorf, 20246 Hamburg, Germany; g.heydecke@uke.de

**Keywords:** oral health, periodontal disease, food and nutrition, diet, inflammation, cross-sectional studies

## Abstract

While the effects of dietary patterns on cardiovascular risk and diabetes have been well studied, the evidence is scarce as to which diet has the greatest anti-inflammatory potential and how dietary patterns are associated with periodontitis. In the Hamburg City Health Study (HCHS), we developed an anti-inflammatory dietary score using a data-driven approach based on the relationship of relevant selected food groups with inflammatory biomarkers (hsCRP and IL-6). The aim of this cross-sectional study was to evaluate the association between the anti-inflammatory dietary score and the incidence of periodontitis in Hamburg, Germany. A total of *n* = 5642 participants fit the required inclusion criteria and were selected for analysis. Periodontal disease was assessed using probing depth, gingival recession, and bleeding on probing. Dietary intake was measured using a food frequency questionnaire (FFQ). A self-developed anti-inflammatory dietary score served as the key explanatory variable. Higher scores reflected lower inflammatory processes (measured through the biomarkers hsCRP and IL-6). Several covariates were included in the regression analysis. Regressions revealed that a higher anti-inflammatory dietary score was significantly associated with lower odds to be affected by periodontal disease in an unadjusted model (OR 0.86, 95% CI 0.82–0.89, *p* < 0.001) and in an adjusted model (age, sex, smoking, diabetes, hypertension, and physical activity) (OR 0.93, 95% CI 0.89–0.98, *p* = 0.003). Our study demonstrated a significant inverse association between an anti-inflammatory dietary score and periodontitis. Individuals with higher intake of proinflammatory nutrition should be specifically addressed to avoid periodontitis.

## 1. Introduction

Periodontitis is an inflammatory disease of the tooth surrounding tissues, which affects up to 11.5 million people in Germany [1]. It is one of the most prevalent oral diseases in the world and a major cause of tooth loss [2]. Additionally, periodontitis is responsible for impacting patients’ quality of life and has been linked to other systemic diseases [3,4]. The destruction of the periodontal tissue originates from an inflammation of the gingiva, which over time may trigger a dysbiosis of the oral microbiota brought about by the colonization of periodontopathogenic bacterial species [5,6,7]. As a result, this causes an interaction with the host’s immune defenses, leading to inflammation, characterized by the release of proinflammatory mediators such as interleukin-6 (IL-6) and high-sensitivity C-reactive protein (hsCRP), which play a major role in the development of chronic periodontitis. Additionally, the host immune response is modulated by various risk factors, such as genetic, behavioral, and environmental [8] factors that need to be understood in order to aid prevention of periodontal disease at the population level. Nutrition is one of the main lifestyle-related factors, which, through the anti- and proinflammatory potential of foods, may modulate the inflammatory process. Proinflammatory diets have been associated with a variety of chronic conditions, such as cancer [9,10] and cardiovascular disease [11,12]. Although the literature on nutrition and oral inflammation is relatively scarce [13,14], studies have shown that a proinflammatory diet also has a significant impact on the pathophysiology of periodontal disease [15,16,17]. With regard to dietary recommendations, the Dietary Approaches to Stop Hypertension (DASH) [18], the Mediterranean diet [19], and the Mediterranean-DASH Intervention for Neurodegenerative Delay (MIND) [20] have been proposed to promote a healthier lifestyle and to reduce the risk of disease [21,22,23,24,25,26]. In our recently published cross-sectional study, we found a significant association between higher adherence to the DASH and the Mediterranean diet and lower odds to be affected by periodontal disease among participants of the Hamburg City Health Study (HCHS) [27].

Yet, evidence as to which food groups have the greatest proinflammatory and anti-inflammatory potential and how these are associated with periodontitis has not been firmly established. Ideally, such a classification should be derived from the investigation of associations with eating patterns across different populations. In the HCHS, we developed an anti-inflammatory dietary score based on the relationship of relevant selected food groups with inflammatory biomarkers (hsCRP and IL-6). Potentially anti- and proinflammatory food groups were both considered. Our aim was to evaluate the association between the anti-inflammatory dietary score and the incidence of periodontitis in the older population in Hamburg, Germany. Based on this, recommendations can be derived regarding the consumption of these foods.

## 2. Materials and Methods

### 2.1. Subjects, Study Design, and Setting

We based our findings on data from the HCHS—an ongoing population-based, prospective cohort study conducted at the University Medical Center Hamburg-Eppendorf. The HCHS aims to collect health-related data from 45,000 citizens aged 45–75 years from the greater area of Hamburg, Germany to gather information regarding disease burden of the older northern population [28]. Baseline data were available for the first 10,000 participants recruited between February 2016 and November 2018. The local ethics committee of the Landesärztekammer Hamburg (State of Hamburg Chamber of Medical Practitioners, PV5131) approved the study protocol and all participants provided informed consent. This study is registered at ClinicalTrial.gov (NCT03934957). In total, *n* = 5642 participants fit the required inclusion criteria and were selected for analysis.

### 2.2. Definition of an Anti-Inflammatory Dietary Score

We used a validated food-frequency questionnaire (FFQ), developed for the European Prospective Investigation into Cancer and Nutrition (EPIC) study, to collect data on the dietary intake of the participants [29]. It records information on frequency and portion size of 102 food items, single nutrients, and supplements that were consumed in the previous year. Subsequently, the collected information was assessed in terms of energy intake, relevant food groups, and nutrients.

As no established anti-inflammatory diet was available, we defined the adherence to an anti-inflammatory diet using a data-driven approach based on our own data on established dietary pattern scores. All items of the Mediterranean, DASH, and MIND diets were considered for the composition of the anti-inflammatory diet. Firstly, adherence to the Mediterranean diet was measured using a validated German version of the original Mediterranean Diet Adherence Screener (MEDAS) [30]. This version assigns scores of 0 or 1 to 12 food items and 2 food intake habits, resulting in a total score ranging from 0 (no adherence) to 14 (perfect adherence). Adherence to the DASH diet was evaluated using a published scoring scheme [31] that assigned scores of 0, 0.5, or 1 to 10 items. Adherence to the MIND diet was calculated, as proposed in the original article [24], with scores of 0, 0.5, or 1 given to 10 healthy and 5 unhealthy nutritional components, producing a total score ranging from 0 (no adherence) to 15 (perfect adherence). In order to avoid extreme sample size differences among adherence scores, the adherence scores for all items of the DASH, Mediterranean, and MIND diets were converted into tertiles, and their associations with the inflammatory markers hsCRP and IL-6 were evaluated using a Kruskal–Wallis test. The item with the highest effect size was selected when multiple items represented the same food group, and the more general item was chosen when multiple general and specific items represented the same food group.

The following items were significantly correlated with inflammatory markers: 4 items of the DASH diet (vegetables (hsCRP: χ^2^ = 42.9, *p* < 0.001; IL-6: χ^2^ = 59.3, *p* < 0.001), fruits (hsCRP: χ^2^ = 28.83, *p* < 0.001; IL-6: χ^2^ = 35.79, *p* < 0.001), total dairy (hsCRP: χ^2^ = 26.94, *p* < 0.001; IL-6: χ^2^ = 33.02, *p* < 0.001), and nuts/seeds/legumes (hsCRP: χ^2^ = 119.04, *p* < 0.001; IL-6: χ^2^ = 72.22, *p* < 0.001); 8 items of the MIND diet (green leafy vegetables (hsCRP: χ^2^ = 38.83, *p* < 0.001; IL-6: χ^2^ = 44.8, *p* < 0.001), other vegetables (hsCRP: χ^2^ = 43.7, *p* < 0.001; IL-6: χ^2^ = 58.29, *p* < 0.001), berries (hsCRP: χ^2^ = 8.22, *p* < 0.001; IL-6: χ^2^ = 28.06, *p* < 0.001), nuts (hsCRP: χ^2^ = 45.48, *p* < 0.001; IL-6: χ^2^ = 47.45, *p* < 0.001), cheese (hsCRP: χ^2^ = 18.86, *p* < 0.001; IL-6: χ^2^ = 26.92, *p* < 0.001), red and processed meat (hsCRP: χ^2^ = 70.95, *p* < 0.001; IL-6: χ^2^ = 23.32, *p* < 0.001), fast fried food (hsCRP: χ^2^ = 13.76, *p* = 0.001; IL-6: χ^2^ = 4.86, *p* = 0.088), and wine (hsCRP: χ^2^ = 61.52, *p* < 0.001; IL-6: χ^2^ = 62.74, *p* < 0.001); and 6 items of the Mediterranean diet (vegetables (hsCRP: χ^2^ = 39.0, *p* < 0.001; IL-6: χ^2^ = 56.79, *p* < 0.001), fruits (hsCRP: χ^2^ = 13.7, *p* < 0.001; IL-6: χ^2^ = 22.52, *p* < 0.001), red meat (hsCRP: χ^2^ = 38.22, *p* < 0.001; IL-6: χ^2^ = 25.14, *p* < 0.001), sugar sweetened carbonated drinks (hsCRP: χ^2^ = 16.75, *p* < 0.001; IL-6: χ^2^ = 11.69, *p* = 0.001), nuts (hsCRP: χ^2^ = 74.4, *p* < 0.001; IL-6: χ^2^ = 32.1, *p* < 0.001), and tomato sauce (hsCRP: χ^2^ = 7.23, *p* = 0.007; IL-6: χ^2^ = 28.23, *p* < 0.001). Among these, several items were duplicates representing the same food group, of which the one with the largest difference between tertiles was selected. The removal of duplicates resulted in the final version of the anti-inflammatory dietary score containing a total of 9 items (4 items from the DASH diet (vegetables, fruits, total dairy, nuts/seeds/legumes); 3 items from the MIND diet (red meat and processed meat, fast fried foods, wine); and 2 items from the Mediterranean diet (sugar sweetened carbonated drinks, tomato sauce). For the final anti-inflammatory dietary score (0–9 points), both anti- and proinflammatory food items were considered. A higher score reflects a nutritional pattern with lower inflammatory potential.

### 2.3. Periodontal Examination

All participants received a full dental examination (six sites protocol, excluding the third molars), performed by trained and calibrated examiners under supervision of a dentist according to a pre-specified SOP [32,33,34]. The periodontal examination was performed with a standardized periodontal probe (Hu-friedy Mfg. Co., LLC, Chicago, IL, USA). Decisive periodontal parameters included probing depth (mm, 6 sites per tooth) and gingival recession (mm, 6 sites per tooth). Further, the plaque index (in %) was measured according to Silness and Löe [35], and bleeding on probing (BOP) (yes/no per tooth, expressed in % of bleeding sites) was assessed at two sites per tooth. Subsequently, the clinical attachment loss (CAL) (mm) was calculated for each tooth by adding probing depth and recession. Additionally, the Number of Decayed, Missing, and Filled Teeth (DMFT) Index was determined. Then, all participants were categorized in one out of three severity levels of periodontitis based on the following criteria by Eke et al. [36]: (1) no periodontitis/mild periodontitis, (2) moderate periodontitis, and (3) severe periodontitis.

### 2.4. Assessment of Additional Variables

For all participants, the following information on confounding variables was retrieved from the HCHS database: sex, age (in years), education (according to ISCED-97 [37]), body mass index (BMI) (in kg/m^2^), smoking (current smoker), diabetes (positive self-declaration and/or taking medication of the A10 group (insulin and analogues), and/or fasting glucose >126 mg/dL, not fasting glucose >200 mg/dL), hypertension (defined as a systolic blood pressure ≥140 mmHg, a diastolic blood pressure ≥90 mmHg, or the use of hypertension medication), intake of hypertension medication (yes), and lipid lowering medication (yes). HbA1c plasma levels (in %), high-density lipoprotein (HDL) cholesterol (mg/dL), and triglycerides (mg/dL) were determined by standard techniques. Low-density lipoprotein (LDL) cholesterol (mg/dL) was calculated using the Friedewald formula. Participants were also asked about their physical activity (yes, ≥1 h/week) and their total sport hours per week. Nutrition was reported as gram/day and included fiber, protein, fat, carbohydrates, and alcohol. IL-6 (mg/L) and hsCRP (mg/dL) were assessed via established ELISA. Further information on the assessment of biomarkers can be found in the HCHS-based study by Struppek et al. [34].

### 2.5. Statistical Analysis

Descriptive analyses were stratified using the periodontitis severity (Table 1) and anti-inflammatory dietary score (Table 2). Continuous variables are displayed as median and interquartile range (median (IQR)). Categorical variables are displayed as absolute numbers and percentages (*n* (%)). Within-group differences were calculated using the chi-squared test for categorical variables and the Kruskal–Wallis rank sum test for continuous variables. The anti-inflammatory dietary score was dichotomized into “low” and “high” (low (0–4.5 points) vs. high (>4.5 points)). Ordinal logistic regression models were applied to determine the association between periodontitis (dependent variable) and the anti-inflammatory dietary score (independent variable) (Table 3). All statistical analyses were performed using the computing software R (Version 4.0.3) with standard *p*-value < 0.05 for statistical significance.

## 3. Results

### 3.1. Descriptive Analyses

Table 1 displays data from 6209 participants with fully completed periodontal examinations. Among the cohort, 1453 participants had either none or mild periodontitis, 3580 participants had moderate periodontitis, and 1176 participants presented with severe periodontitis. Participants with severe periodontitis were more likely to be older men (median age 66 vs. 59 years and 61% vs. 40% males), with less frequent higher education (42% vs. 49%) compared to participants with none/mild periodontitis. Additionally, participants with severe periodontitis presented more cardiovascular risk factors (BMI, smoking, diabetes, and hypertension), higher overall medication intake, as well as higher inflammatory biomarker levels (IL6 and hsCRP) (1.77 vs. 1.45 and 0.13 vs. 0.10). They also had a higher total energy intake (kcal/day) (2068.9 vs. 1989.2) compared to participants with none/mild periodontitis. Participants with severe periodontitis presented a lower anti-inflammatory dietary score (4.5 vs. 5.0), with higher scores reflecting lower inflammatory processes, and a higher DMFT Index score (21.0 vs. 17.0), plaque index score (22.0 vs. 0.0), and mean CAL (3.3 vs. 1.8) (Table 1). Table 2 displays data from 5642 participants with complete information on the anti-inflammatory dietary score. Participants with low adherence to an anti-inflammatory diet (= low anti-inflammatory dietary score) presented a higher BOP score (8.9 vs. 7.1), higher plaque index score (10.4 vs. 5.4), and were more affected by severe periodontitis (22% vs. 16%) compared to participants with high adherence to an anti-inflammatory diet (=high anti-inflammatory dietary score) (Table 2).

### 3.2. Regression Analysis

The ordinal logistic regression models revealed a significant association between a higher anti-inflammatory dietary score and lower odds to present periodontitis in an unadjusted model (OR 0.86, 95% CI 0.82–0.89, *p* < 0.001) and in the following three stepwise adjusted models: adjusted Model 1 (age, sex) (OR 0.90, 95% CI 0.86–0.94, *p* < 0.001), adjusted Model 2 (age, sex, smoking, diabetes, hypertension) (OR 0.92, 95% CI 0.88–0.97, *p* = 0.001), and adjusted Model 3 (age, sex, smoking, diabetes, hypertension, and physical activity) (OR 0.93, 95% CI 0.89–0.98, *p* = 0.003). The results of the ordinal regressions are given in Table 3.

## 4. Discussion

### 4.1. Main Findings

Using data from the FFQ of the HCHS on dietary patterns of study participants, we developed an anti-inflammatory dietary score. Our aim was to investigate the association between adherence to an anti-inflammatory diet and periodontitis. Regressions revealed a significant association between a higher anti-inflammatory dietary score (higher adherence to an anti-inflammatory diet) and lower odds to present periodontitis. Our study adds to previous knowledge, and is the first study to examine the association between potentially anti- and proinflammatory food groups and periodontitis in an older German population.

### 4.2. Previous Research and Possible Explanations

In our study and various other studies, periodontitis was associated with increased levels of inflammatory biomarkers [38,39,40,41]. Diet is a modifiable factor for systemic inflammation, which plays an important role in the etiology of disease. Proinflammatory elements of diet lead to an increase in circulating interleukin levels. As a result, this leads to an increased production of hsCRP, which plays an important role in systemic inflammation [40]. Systemic inflammation may foster the growth of dysbiotic microbial communities and enhance gingival inflammation, which in response may induce or lead to the progression of periodontitis [40]. For example, improved markers of systemic inflammation were found in a 12-week dietary intervention study with a high-fat, low-carbohydrate diet [42]. However, the authors did not gather clinical oral health parameters of the study participants. In a recent randomized controlled dietary intervention trial, the experimental group was instructed to change to an anti-inflammatory diet for 6 weeks, while the control group was instructed not to change their diet [17]. Prior to the intervention, both groups continued their Western diet for a period of 2 weeks. The anti-inflammatory diet was low in processed carbohydrates and animal proteins and rich in omega-3 fatty acids, vitamin C, vitamin D, antioxidants, plant nitrates, and fibers. At the end of the trial, the experimental group showed a significant reduction in gingival bleeding scores. Inflammatory parameters, however, remained unaffected. The authors attributed this to the relatively short study period. Moreover, the authors reported that switching from 2 weeks of Western diet to an anti-inflammatory diet was very stressful for the participants of the experimental group. The change to a maximum healthy diet and getting used to it occurred in a very short period of time, which caused stress to the participants’ bodies. In consequence, this might have led to the unchanged inflammatory parameters. Had the authors observed the participants for an additional 2–3 months, the inflammatory markers may have been different [17].

Currently, there is no established definition of an anti-inflammatory diet available and proinflammatory nutrition is still poorly understood, especially when it comes to oral health. Nevertheless, other dietary patterns that comprise inflammatory components have been studied. The Mediterranean and DASH diets have both been shown to be associated with a reduction in inflammatory markers [27,43,44,45,46,47] as well as a reduction in periodontopathogenic bacteria [48] and better periodontitis outcomes [27]. All diets recommend a high consumption of plant-based foods (fruits, vegetables, legumes, nuts, and whole grain products) and to avoid the consumption of sweets, highly processed foods, and red meat. Our aim was to develop an anti-inflammatory dietary score based on the relationship of relevant selected food groups with biomarkers of inflammation (hsCRP and IL-6). Thus, we studied the adherence to a diverse set of dietary patterns (DASH, Mediterranean, and MIND diets), whose beneficial health outcomes have been shown in clinical and epidemiological studies [49,50,51,52], and we selected certain food groups that correlated with inflammatory biomarkers. Vegetables, fruits, and nuts/seeds/legumes were identified as anti-inflammatory foods for our score and should be consumed in high quantities. This has also been confirmed in other studies [53]. In a meta-analysis of 29 intervention trials, Eichelmann and colleagues found that consumption of a plant-based diet was associated with a reduction in hsCRP and IL-6 levels [54]. Additionally, in a cross-sectional study on the intake of dietary fiber from different food sources and their associations with measures of body composition and inflammation, fiber from fruits and vegetables or legumes were significantly associated with the reduction of hsCRP levels [55]. Research has shown that the intake of these foods can improve oral health outcomes and can help to prevent the progression of periodontal disease [56,57,58,59]. While the frequent consumption of refined carbohydrates, high-saturated fat, and low-fiber increases the risk of periodontitis [60], studies have demonstrated that diets with foods rich in antioxidants, minerals, vitamins, omega-3 polyunsaturated fatty acids, and fibers benefit the health of periodontal tissues [16,57,61,62,63,64]. For example, the MIND diet differentiates itself from other dietary patterns by including green leafy vegetables, separate from other vegetables, which contain a lot of antioxidants and have been proven to be beneficial for oral health outcomes [20]. We found that this food group was significantly inversely associated with hsCRP and IL-6 levels. On the other hand, red meat, processed meat, and fast fried foods were associated with higher hsCRP and IL-6 levels. The intake of these foods should therefore be limited or avoided. In a randomized controlled feeding trial from China, participants randomized to consume fried meat four times per week for one month demonstrated significantly increased systemic inflammation compared to the control group [65]. Significantly positive associations between red and processed meat consumption and hsCRP levels have also been found in other studies [66,67,68]. Another food group that was significantly associated with higher hsCRP and IL-6 levels is sugar sweetened carbonated drinks. Regular consumption of these drinks has been associated with increased biomarkers of inflammation [69,70,71,72,73] and has been studied in association with a variety of diseases [74,75,76,77,78]. Sugar sweetened carbonated drinks, which often are consumed between meals without rinsing or tooth brushing afterwards, have been associated with increased risk of periodontal disease [79,80,81,82] as well as dental caries and tooth erosion [83,84,85,86]. The consumption of processed meat, fried foods, and sugar sweetened carbonated drinks is very common in the Western diet [60] that is highly prevalent in Germany [87,88]. Hence, references for the consumption of these foods should not be neglected in dietary recommendations. Importantly, the anti-inflammatory dietary score considers both anti- and proinflammatory foods.

The literature-derived, population-based dietary inflammatory index (DII) [89], which characterizes the inflammatory potential of diet and links it to a food intake database, has been applied in a large variety of studies with respect to risk of cardiac and metabolic disease [90,91]. In terms of oral health, the DII has been associated with fewer missing teeth [92] and lower mean values of periodontal parameters [15,93]. The DII and the empirical dietary inflammatory pattern (EDIP) [94] have both been associated with higher concentrations of inflammatory markers [95]. The EDIP was developed in the Nurses’ Health Study, an ongoing prospective cohort of female registered nurses aged 30–55 years at enrollment in 1976 [96]. The score is based on well-educated health professionals within the United States. Due to the potential homogeneity in dietary exposure, this, however, might present problems with respect to the comparability to other populations. In a prospective cohort study among male health professionals, no association between EDIP and incidence of self-reported periodontitis was reported [97]. The DII is mainly based on nutrients, and the literature on which it is based tested the effect of dietary parameters one at a time. The inflammatory potential of diet, however, requires a more comprehensive view rather than a single-nutrient based assessment. For example, dietary patterns that take multiple dietary factors into account provide a more comprehensive assessment of diet, and therefore have a better predictive value for associations between diet and disease.

It is important to note that the DASH, Mediterranean, and MIND diets were not specifically developed for the HCHS study population. Thus, the dietary patterns might not fully reflect the dietary behaviors of the target population. Findings of certain dietary patterns differ across populations [98]. For example, the Mediterranean diet recommends foods that are commonly consumed in Mediterranean countries, such as Italy or Greece. Due to variations in the availability of these food items in other countries and regions, the transferability of the diet might be difficult [99]. Nevertheless, each of the studied dietary patterns consists of different food items that may play a role in the modulation of the host’s inflammatory response by reducing inflammatory biomarkers. By incorporating all of them, we ensured the specification of the nutrition in its entirety. Although, it would be important to consider synergistic effects of specific micro- and macronutrients and their effects on inflammation and oral health. At the population level, dietary recommendations of specific anti-inflammatory food items and food groups appear to be more implementable and comprehensible instead of nutrient-based recommendations.

Considering that we use our teeth to chew our foods and that the mouth, or oral cavity, is where the digestive system begins, it seems logical that our dietary habits play an important role in our periodontal health. We could, however, also consider how our periodontal health might affect our dietary habits. Periodontitis, if untreated, can lead to the loss of teeth [100]. As a consequence, it has been shown that tooth loss in adults might influence dietary intake. Poor dentition is associated with lower diet quality, a decline in dietary variety, and reduced intake of important nutrients [101,102,103]. Adults with poor dentition might find it difficult to chew harder foods, such as certain fruits, vegetables, or whole grain, which are a source of important nutrients [104].

### 4.3. Strengths and Limitations

Our study has some limitations. The population of the HCHS is middle-aged white Caucasian from Northern Germany. Hence, our findings may not be generalized to other ethnicities, regions, or to a much younger population. Furthermore, the cross-sectional nature of our study does not allow for any inference of causality between periodontitis and our anti-inflammatory dietary score. Additionally, a further limitation is that with self-reported dietary intake, some measurement error is to be expected. Yet, the FFQ appears to be a reasonably valid tool for the assessment of dietary intake among German adults [105,106]. For future research, it would be interesting to include information on other inflammation markers, such as other interleukins and TNF-α, and to further consider synergistic effects of specific nutrients and their effect on inflammation.

Nevertheless, there are a number of strengths that are worth mentioning. The large sample size is based on data from the HCHS and representative of the older German adult population. We conducted a full periodontal examination and examiners were calibrated, which increases the validity and accuracy of the results. Moreover, a variety of covariates that could potentially affect the association were included in our analyses, therefore ensuring the comprehensiveness and consistency of study results.

## 5. Conclusions

Based on a data-driven approach, we used sub-items (both anti- and proinflammatory foods) from other dietary patterns to define an anti-inflammatory dietary score, with a focus on the inflammatory potential of certain food groups. Our study shows a significant association between a proinflammatory diet and periodontitis. Therefore, we hypothesize that the consumption of a proinflammatory diet may aggravate the clinical symptoms of periodontitis patients. Nevertheless, whether or not an anti-inflammatory diet might help in the regression of disease, warrants the implementation of randomized controlled trials. In addition to periodontal therapy and emphasizing appropriate oral hygiene routines, dental professionals could provide dietary recommendations to their patients. However, most dentists focus on making recommendations about limiting sugar consumption or ask about exposure to cavity-producing foods, especially for children/adolescents [107]. Recommendations on which foods are proinflammatory and anti-inflammatory are rarely discussed. One factor is that nutritional medicine is not part of the curriculum of dental studies [108]. Hence, we suggest that nutrition should be incorporated into the training of dental health professionals and that additional focus should be put on assessing the dietary patterns and behaviors of patients to specifically recommend anti-inflammatory foods for better periodontal outcomes. The anti-inflammatory dietary score could potentially be used to guide individuals in setting dietary goals to help to reduce levels of inflammation, which could be a cost-effective approach for improving population oral health.

## Figures and Tables

**Table 1:** Demographic and clinical characteristics of the participants.

Characteristics	Overall, *N* = 6209 ^1^	Periodontitis			*p*-Value ^2^
		None/Mild *N* = 1453 (23%) ^1^	Moderate *N* = 3580 (58%) ^1^	Severe *N* = 1176 (19%) ^1^	
Sociodemographics					
Sex					<0.001
Male	3057 (49%)	575 (40%)	1766 (49%)	716 (61%)	
Female	3152 (51%)	878 (60%)	1814 (51%)	460 (39%)	
Age (in years)	62.0 (55.0,69.0)	59.0 (52.0,66.0)	63.0 (55.0, 69.0)	66.0 (59.0, 71.0)	<0.001
Education					<0.001
Low	269 (4.5%)	45 (3.2%)	164 (4.8%)	60 (5.4%)	
Medium	2921 (49%)	660 (47%)	1673 (49%)	588 (53%)	
High	2726 (46%)	686 (49%)	1579 (46%)	461 (42%)	
(Missing)	293	62	164	67	
Risk factors					
BMI (kg/m^2^)	26.0 (23.5, 29.0)	25.6 (23.0, 28.7)	26.0 (23.5, 29.0)	26.4 (24.1, 29.7)	<0.001
(Missing)	321	83	170	68	
Smoking (current)	1136 (18%)	235 (16%)	608 (17%)	293 (25%)	<0.001
(Missing)	34	5	22	7	
Diabetes (yes)	449 (7.8%)	85 (6.2%)	242 (7.4%)	122 (11%)	<0.001
(Missing)	464	75	294	95	
Hypertension (yes)	3844 (65%)	768 (55%)	2266 (66%)	810 (72%)	<0.001
(Missing)	274	52	164	58	
Biomarker					
IL6 (mg/L)	1.57 (1.14, 2.20)	1.45 (1.01, 2.04)	1.55 (1.15, 2.16)	1.77 (1.33, 2.63)	<0.001
(Missing)	3014	682	1746	586	
hsCRP (mg/dL)	0.120 (0.060, 0.250)	0.100 (0.060, 0.230)	0.110 (0.060, 0.250)	0.130 (0.070, 0.300)	<0.001
(Missing)	374	92	222	60	
LDL cholesterol (mg/dL)	121.0 (96.0, 146.0)	118.0 (96.0, 145.5)	122.0 (97.0, 145.0)	121.0 (93.8, 146.0)	0.161
(Missing)	237	46	143	48	
HDL Cholesterol (mg/dL)	85.0 (75.0, 97.0)	87.0 (77.0, 99.0)	85.0 (75.0, 97.0)	84.0 (74.0, 97.0)	<0.001
(Missing)	237	46	143	48	
Medication intake					
Hypertension medication (yes)	1890 (32%)	371 (26%)	1109 (33%)	410 (37%)	<0.001
(Missing)	264	34	169	61	
Lipid lowering Medication Lipid (yes)	1014 (17%)	200 (14%)	567 (17%)	247 (22%)	<0.001
(Missing)	264	34	169	61	
Dietary parameters					
Total energy (kcal/day)	2035.1 (1617.0, 2582.0)	1989.2 (1568.7, 2543.3)	2047.6 (1628.7, 2593.7)	2068.9 (1639.3, 2602.3)	0.057
(Missing)	566	125	316	125	
Anti-inflammatory dietary score (Score 0–9 points)	4.5 (4.0,5.5)	5.0 (4.0,6.0)	5.0 (4.0,5.5)	4.5 (3.5,5.5)	<0.001
(Missing)	567	126	316	125	
Physical parameters					
Physical activity (yes, ≥1 hour/week)	3926 (72%)	983 (75%)	2286 (72%)	657 (65%)	<0.001
(Missing)	733	151	422	160	
Sport hour per week	2.0 (0.0, 4.0)	2.0 (0.4, 4.0)	2.0 (0.0, 4.0)	2.0 (0.0, 3.5)	<0.001
(Missing)	733	151	422	160	
Dental parameters					
DMFT Index	19.0 (15.0, 23.0)	17.0 (14.0, 21.0)	19.0 (16.0, 23.0)	21.0 (17.0, 24.2)	<0.001
BOP Index (%)	7.7 (1.9, 20.4)	2.1 (0.0, 7.1)	8.3 (2.2, 19.2)	21.1 (9.3, 41.7)	<0.001
(Missing)	101	47	51	3	
Plaque index (%)	7.9 (0.0, 27.8)	0.0 (0.0,10.7)	8.9 (0.0, 27.8)	22.0 (5.8, 54.8)	<0.001
(Missing)	86	8	53	25	
Mean CAL (mm)	2.4 (2.0, 2.8)	1.8 (1.6, 2.1)	2.4 (2.2, 2.7)	3.3 (2.9, 3.9)	<0.001
(Missing)	5	1	3	1	
Sites/mouth CAL ≥ 3 mm (%)	36.5 (19.8, 57.4)	12.5 (6.5,20.8)	38.7 (25.6, 53.6)	68.5 (53.2, 83.3)	<0.001
(Missing)	5	1	3	1	

^1^ Median (IQR) for continuous, *n* (%) for categorical; ^2^ Pearson’s chi-squared test, Kruskal–Wallis rank sum test.

**Table 2:** Dental characteristics of the participants, stratified by anti-inflammatory dietary score.

Characteristics	Overall, *N* = 5642 ^1^	Anti-Inflammatory Dietary Score		*p*-Value ^2^
		Low *N* = 2838 (50%) ^1^	High *N* = 2804 (50%) ^1^	
Dental parameters				
DMFT Index	19.0 (15.0, 23.0)	19.0 (16.0, 23.0)	19.0 (15.0, 22.0)	<0.001
BOP Index (%)	7.7 (1.9, 20.0)	8.9 (2.1, 21.4)	7.1 (1.9, 18.5)	<0.001
(Missing)	78	29	49	
Plaque index (%)	7.7 (0.0, 27.1)	10.4 (0.0, 32.1)	5.4 (0.0, 22.0)	<0.001
(Missing)	80	40	40	
Mean CAL (mm)	2.4 (2.0, 2.8)	2.4 (2.1, 2.9)	2.3 (2.0, 2.7)	<0.001
(Missing)	2	0	2	
Sites/mouth CAL ≥ 3 mm (%)	36.3 (19.6, 57.1)	38.6 (20.8, 60.5)	34.3 (18.5, 53.2)	<0.001
(Missing)	2	0	2	
Periodontitis				<0.001
None/mild	1327 (24%)	607 (21%)	720 (26%)	
Moderate	3264 (58%)	1619 (57%)	1645 (59%)	
Severe	1051 (19%)	612 (22%)	439 (16%)	

^1^ Median (IQR) for continuous, *n* (%) for categorical; ^2^ Wilcoxon rank sum test, Pearson’s chi-squared test; anti-inflammatory dietary score = low (0–4.5 points) vs. high (>4.5 points).

**Table 3:** Ordinal logistic regression for the association between the anti-inflammatory dietary score and periodontitis (outcome: periodontitis).

	Model 1			Model 2			Model 3			Model 4		
Predictors	Odds Ratios	CI	*p*	Odds Ratios	CI	*p*	Odds Ratios	CI	*p*	Odds Ratios	CI	*p*
Anti-inflammatory dietary score	0.86	0.82–0.89	**<0.001**	0.90	0.86–0.94	**<0.001**	0.92	0.88–0.97	**0.001**	0.93	0.89–0.98	**0.003**
Age				1.05	1.04–1.06	**<0.001**	1.05	1.04–1.06	**<0.001**	1.05	1.04–1.06	**<0.001**
Sex (female)				0.67	0.60–0.75	**<0.001**	0.68	0.60–0.76	**<0.001**	0.68	0.60–0.76	**<0.001**
Smoking (yes)							1.67	1.44–1.94	**<0.001**	1.65	1.42–1.92	**<0.001**
Diabetes (yes)							1.04	0.84–1.29	0.702	1.00	0.80–1.24	0.978
Hypertension (yes)							1.21	1.07–1.37	**0.002**	1.25	1.11–1.42	**<0.001**
Physical activity (yes)										0.92	0.81–1.04	0.196

Ordinal logistic regression model with outcome = periodontitis. Adjusted for: age, sex, smoking, diabetes, hypertension, and physical activity. Male sex serves as a reference and are not displayed in the table.

## Data Availability

Restrictions apply to the availability of these data. Data were obtained from the Hamburg City Health Study and are available from the authors with the permission of the Hamburg City Health Study.
